# The Late Sir Arthur Pearson

**Published:** 1922-01

**Authors:** Neville A. Pearson


### CORRESPONDENCE.

[Correspondence on all subjects is invited, but we cannot in any way be responsible for the opinions expressed by our correspondents, who must give their name and address as a guarantee of good faith, but not necessarily for publication. Correspondents are reminded that brevity of style and conciseness of statement greatly facilitate early insertion.]

### The Late Sir Arthur Pearson.

*To the Editor of* THE HOSPITAL AND HEALTH REVIEW.

DEAR SIR,—At this moment of my own and my mother's deep personal loss, I feel sure that I am voicing what would have been my father's wishes in asking that the public who have so fully and generously supported his unceasing work for St. Dunstan's and the National Institute for the Blind, should continue to help that work in every possible way.

My father's deepest concern was that the public should regard his work for the blind not merely as a personal effort of his own, but as a National Trusteeship placed in his hands by the people of the Empire.

I ask, therefore, in his name, that all present and projected activities to raise the much needed funds for the work to which my father devoted his life, should continue without ceasing for a moment. I am sure that he would have wished this.

Believe me, Dear Sir,
Yours sincerely,
NEVILLE A. PEARSON.

